# Appendicitis

**Published:** 1897-09

**Authors:** 


### APPENDICITIS.

Notwithstanding claims to the contrary, twenty cases of appendicitis to one are cured permanently without operation. —*Professor Wm. Pepper, formerly Provost of the University of Pennsylvania.*

